# Fluorescence Assay for Detecting Four Organophosphorus Pesticides Using Fluorescently Labeled Aptamer

**DOI:** 10.3390/s22155712

**Published:** 2022-07-30

**Authors:** He Li, Xue Huang, Jingcheng Huang, Mengyuan Bai, Mengjiao Hu, Yemin Guo, Xia Sun

**Affiliations:** 1 School of Agricultural Engineering and Food Science, Shandong University of Technology, No. 266 Xincun Xilu, Zibo 255049, China; sdlihe88@163.com (H.L.); hxxh1985@163.com (X.H.); 17864386069@163.com (J.H.); bai15695430950@163.com (M.B.); hu17852034056@163.com (M.H.); gym@sdut.edu.cn (Y.G.); 2 Shandong Provincial Engineering Research Center of Vegetable Safety and Quality Traceability, No. 266 Xincun Xilu, Zibo 255049, China; 3 Zibo City Key Laboratory of Agricultural Product Safety Traceability, No. 266 Xincun Xilu, Zibo 255049, China

**Keywords:** aptamer, tetramethylrhodamine, guanine, fluorescence assay, organophosphorus pesticide

## Abstract

In this work, we reported a rapid and sensitive fluorescence assay in homogenous solution for detecting organophosphorus pesticides by using tetramethylrhodamine (TAMRA)-labeled aptamer and its complementary DNA (cDNA) with extended guanine (G) bases. The hybridization of cDNA and aptamer drew TAMRA close to repeated G bases, then the fluorescence of TAMRA was quenched by G bases due to the photoinduced electron transfer (PET). Upon introducing the pesticide target, the aptamer bound to pesticide instead of cDNA because of the competition between pesticide and cDNA. Thus, the TAMRA departed from G bases, resulting in fluorescence recovery of TAMRA. Under optimal conditions, the limits of detection for phorate, profenofos, isocarbophos, and omethoate were 0.333, 0.167, 0.267, and 0.333 µg/L, respectively. The method was also used in the analysis of profenofos in vegetables. Our fluorescence design was simple, rapid, and highly sensitive, which provided a means for monitoring the safety of agricultural products.

## 1. Introduction

In order to control diseases and pests, organophosphorus pesticides (OPs), such as phorate, profenofos, isocarbophos, and omethoate, have been introduced into agricultural production for many years [1,2]. Most OPs have acute and residual toxicity, and they can cause serious pollution to soil, water, and even food due to their widespread abuse in various places of the world [3]. As a result, they pose a serious threat to human health by disrupting the neurotransmitter acetylcholine (ACh) in human bodies [4,5]. Thus, it is very necessary to develop a rapid and highly sensitive detection method for OPs residues in vegetables and fruits. In the European Union, the maximum residue limits (MRLs) of organophosphorus pesticides in agricultural products generally range from 0.01 to 0.4 mg/kg. Some high-precision analytical tools have been used for the detection of pesticides including OPs, such as mass spectrometry (MS), high-performance liquid chromatography (HPLC), gas chromatography (GC), and so on [6,7,8,9,10]. However, due to their shortcomings, such as complex operation, high cost, and long time-consumption, the research of a simple, rapid, stable detection technology for OPs is an urgent need. 

Aptamers are single-stranded DNA or RNA, which can bind with the corresponding ligands with high affinity and strong specificity. Aptamer-based biosensors have received more and more attention in target recognition due to the advantages of aptamer [11,12,13], such as easy synthesis and modification, high affinity, good chemical stability, low cost, and non-toxicity [14,15,16,17]. Various aptamer-based assays, such as electrochemiluminescence [18,19], electrochemistry [20,21,22], colorimetry [23,24], and fluorescence [25,26], have been developed for detecting toxic residues in food. Among these methods, fluorescence analysis is especially characterized by fast speed, high sensitivity, convenient operation, and good practicability.

Electron transfer occurs between fluorophore and quencher when they approach, resulting in fluorescence quenching of fluorophore. This is a mechanism of photoinduced electron transfer (PET). We know that tetramethylrhodamine–guanine (TAMRA–G) interaction will limit the local rotation of TAMRA, and quench its fluorescence through PET [27,28,29]. This TAMRA–G interaction can be used for fluorescence analyses, where TAMRA-labeled aptamers are used as fluorescent probes. At present, fluorescence signal studies based on competitive binding, and researches on fluorescence anisotropy/polarization of TAMRA have been reported [30,31,32]. However, there is no report on G quenching the fluorescence of TAMRA for detecting OPs in food.

Herein, taking advantage of aptamer and fluorescence analysis, using TAMRA-labeled aptamer and its complementary DNA with G bases extension, we presented a rapid and sensitive detection method for four OPs (phorate, profenofos, isocarbophos, and omethoate). After the experimental conditions were optimized, the relationships between the fluorescence signal of TAMRA and the concentration of OPs were studied and calculated. Moreover, the selectivity and practicability of this fluorescence method were also tested.

## 2. Experimental

### 2.1. Materials

Pesticides, such as phorate, profenofos, isocarbophos, omethoate, etc., were provided by Shanghai Pesticide Research Institute Co., Ltd. (Shanghai, China). Vegetables came from our planting base (Zibo, China). All other chemicals and reagents were analytically pure. All aqueous solutions were prepared with ultrapure water obtained from a German Milli-Q water purification system. All the DNA oligonucleotides (Table 1) were synthesized and purified (by HPLC) by Sangon Biotech (Shanghai, China). The aptamer we selected had the characteristics of strong specificity and high sensitivity [11,20,33]. The sequence of the aptamer with TAMRA labeled on the 5′ terminal was named TAMRA-Apt, the complementary DNA with a total of 15 bases was named cDNA15, and we called cDNA15 with four guanine bases (optimized) extension at the 3′ end as cDNA15-G4.

### 2.2. Experimental Measurements

Fluorescence analysis was performed on a fluorescence spectrophotometer (RF-6000, Japan) with an excitation at 558 nm and an emission at 583 nm. Without other statement, binding buffer contained 10 mM Tris-HCl (pH 7.5), 1 mM EDTA, 50 mM MgCl_2_, 50 mM NaCl, and 0.01% Tween-20.

Fluorescence assays were carried out with the solution containing 20 nM TAMRA-Apt (optimized), 60 nM cDNA15-G4 (optimized), and different concentrations of OPs in the binding buffer. The solution was incubated at 25 °C for 60 min (optimized). Then, fluorescence intensity of the reaction mixture was measured. Three measurements were made for each sample and the average value was used.

## 3. Results and Discussion

### 3.1. Principle of the Fluorescence Assay

**Scheme 1:**
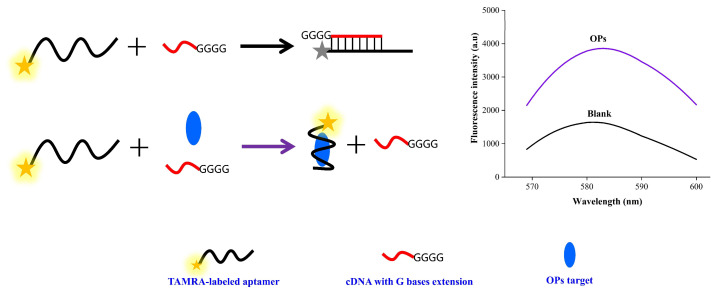
Principle of fluorescence assay for detecting OPs in homogeneous solution by using TAMRA-labeled aptamer and its complementary DNA (excitation wavelength was 558 nm).

As Scheme 1 shows, the 5′ terminal of aptamer is marked with TAMRA, and the 3′ end of cDNA has G bases added. In the absence of OPs target, hybridization between aptamer and cDNA occurs by complementary base pairing. TAMRA and G bases are in close contact with each other, then the TAMRA–G interaction causes fluorescence quenching of TAMRA through photoinduced electron transfer (PET) [27,34]. However, once the OPs target is present, aptamer binds to OPs instead of cDNA due to their competition, then TAMRA–G interaction is broken and the fluorescence of TAMRA recovers. Thus, fluorescence intensity of TAMRA label in duplex increases with addition of OPs, the detection of which can be obtained by observing the fluorescence signal.

### 3.2. Feasibility of the Fluorescence Assay

**Figure 1:**
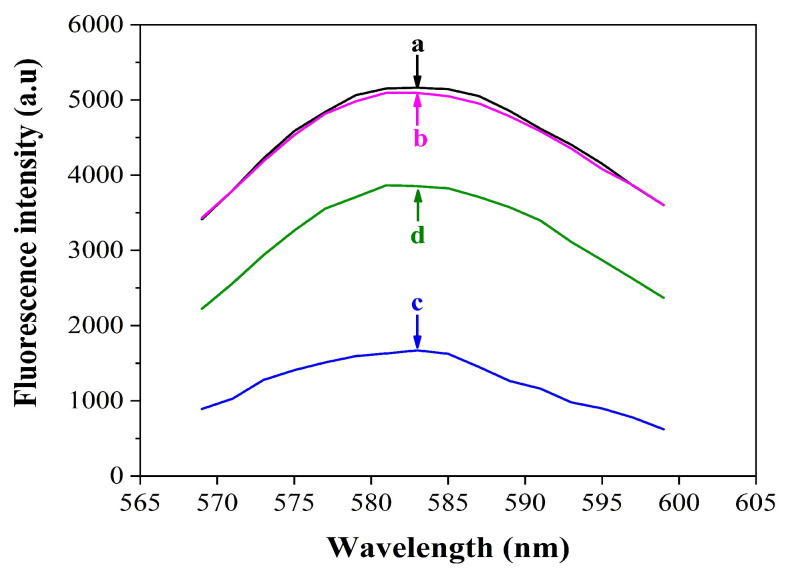
Fluorescence spectrums of different systems (excitation wavelength was 558 nm). (**a**) 20 nM TAMRA-Apt, (**b**) mixture of 20 nM TAMRA-Apt and 60 nM cDNA15, (**c**) mixture of 20 nM TAMRA-Apt and 60 nM cDNA15-G4, and (**d**) mixture of 20 nM TAMRA-Apt, 60 nM cDNA15-G4, and 100 µg/L OPs. All samples were incubated at 25 °C for 60 min.

Firstly, we verified the feasibility of the proposed fluorescence assay. As Figure 1a showed, the excitation wavelength was set to 558 nm, the fluorescence intensity at 583 nm (expressed by FI, the same below) of free TAMRA-Apt was 5160. When the cDNA15 with no extended G bases was added to this system, the FI value of the duplex that TAMRA-Apt and cDNA15 hybridized was 5099 (Figure 1b), which was not much different from free TAMRA-Apt. On the contrary, when cDNA15 was replaced by cDNA15-G4 that contained extended G bases at the end, the FI value of the duplex of TAMRA-Apt and cDNA15-G4 was 1671 (reduced by 68%) (Figure 1c). This showed that extended G bases in cDNA could significantly quench the fluorescence of TAMRA in duplex. When a certain concentration (100 µg/L) of OPs target was added to the system of TAMRA-Apt and cDNA15-G4, the FI value increased from 1671 to 3865 (increased by 131%) (Figure 1d). It indicated that our proposed fluorescence assay was feasible for the detection of OPs.

### 3.3. Optimizations

**Figure 2:**
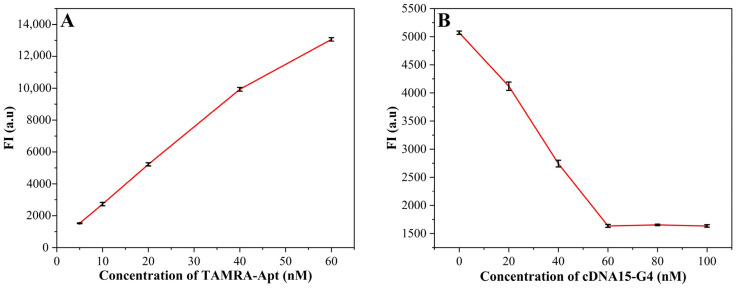
Concentration dependent fluorescence response of TAMRA-Apt (**A**) and concentration dependent quenching efficiency of cDNA15-G4 (TAMRA-Apt concentration: 20 nM, quenching time: 60 min) (**B**).

Experimental conditions strongly affected the performance of the proposed fluorescence assay, so we investigated and optimized them carefully. The fluorescence responses of TAMRA-Apt at different concentrations were obtained. It was showed in Figure 2A that with increasing TAMRA-Apt concentration, the FI value also improved. When the concentration of TAMRA-Apt was 20 nM, we could obtain enough fluorescence to carry out this fluorescence assay. Similarly, the quenching performance gradually increased with increasing cDNA15-G4 concentration, 60 nM or greater concentration could quench the fluorescence of TAMRA-Apt (20 nM) to the maximum extent, about 68% quenching was observed at 60 nM (Figure 2B). Therefore, 20 nM TAMRA-Apt and 60 nM cDNA15-G4 were selected for this fluorescence assay.

**Figure 3:**
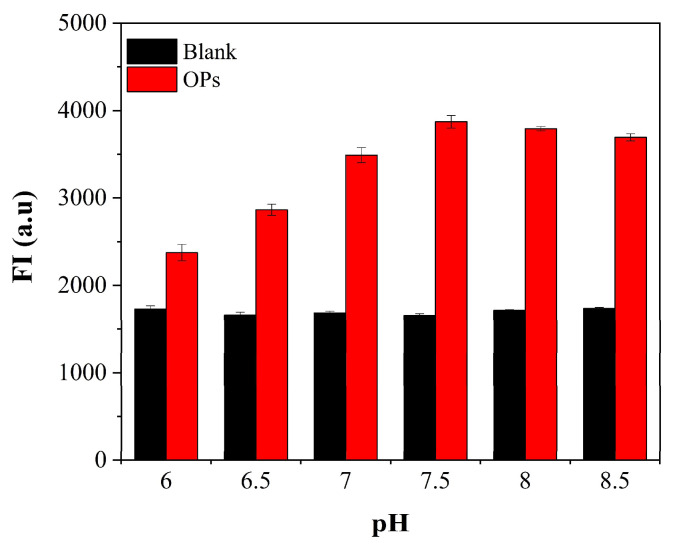
Effect of buffer pH on fluorescence recovery caused by OPs (100 µg/L) using TAMRA-Apt (20 nM) and cDNA15-G4 (60 nM) (incubation temperature: 25 °C, incubation time: 60 min).

Buffer pH was an important factor that could affect the performance of this assay. It could be observed in Figure 3 that with increasing pH from 6.0 to 7.5, the FI recovery caused by OPs gradually improved. When the pH was greater than 7.5, the FI recovery slightly reduced. Therefore, the buffer with a pH of 7.5 was chosen in this assay because a large change in fluorescence signal was caused by OPs.

**Figure 4:**
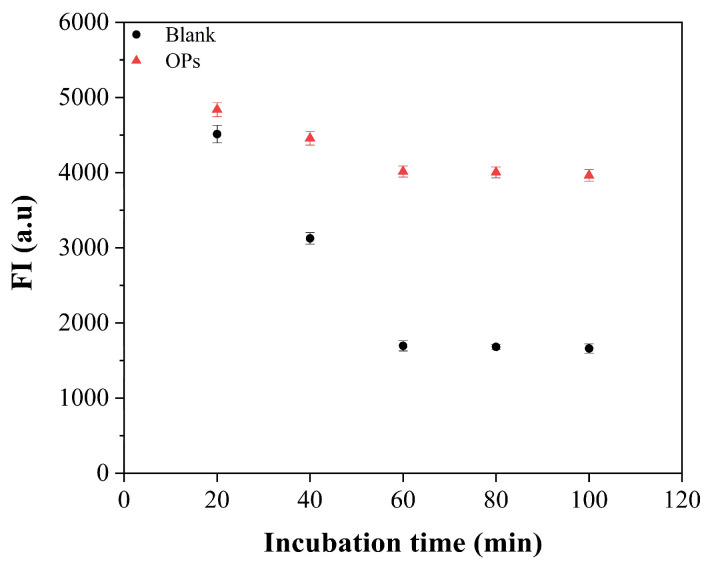
Effect of incubation time on fluorescence intensity of TAMRA-Apt (20 nM) and cDNA15-G4 (60 nM) in the absence and presence of OPs (100 µg/L) (buffer pH: 7.5, incubation temperature: 25 °C).

Incubation time played an important role in our assay. As time went on, the quenching effect of cDNA15-G4 on TAMRA-Apt gradually increased (Figure 4). Finally, fluorescence determination was performed after 60 min of incubation because a large OPs-induced FI recovery was obtained.

**Figure 5:**
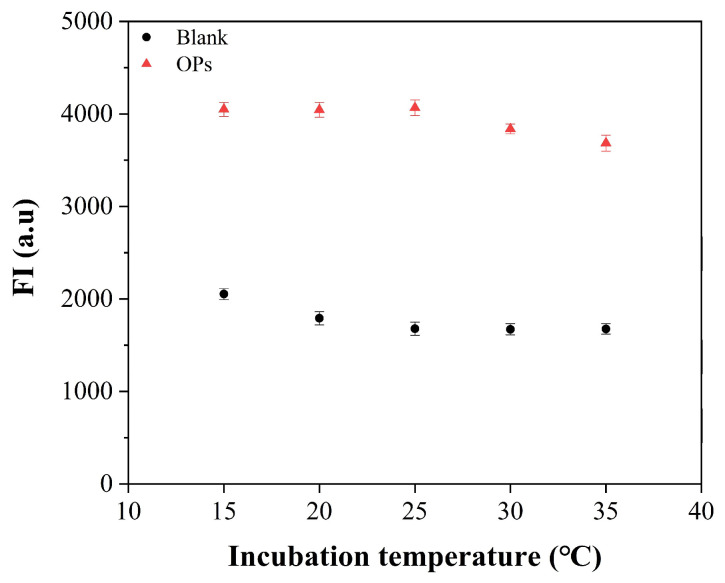
Effect of incubation temperature on fluorescence recovery caused by OPs (100 µg/L) using TAMRA-Apt (20 nM) and cDNA15-G4 (60 nM) (buffer pH: 7.5, and incubation time: 60 min).

The incubation temperature was also an important factor that could significantly affect the fluorescence response in this assay. Figure 5 showed that the quenching efficiency of cDNA15-G4 on TAMRA-Apt enhanced with the increasing incubation temperature from 15 °C to 25 °C and then leveled off. In the end, due to the large signal change caused by OPs, 25 °C was chosen as the optimal incubation temperature. It could also be seen that when the incubation temperature was above 25 °C, the OPs-induced FI recovery slightly reduced. This might be caused by the inactivation of the aptamer under high temperature.

### 3.4. Quantitative Measurement

**Figure 6:**
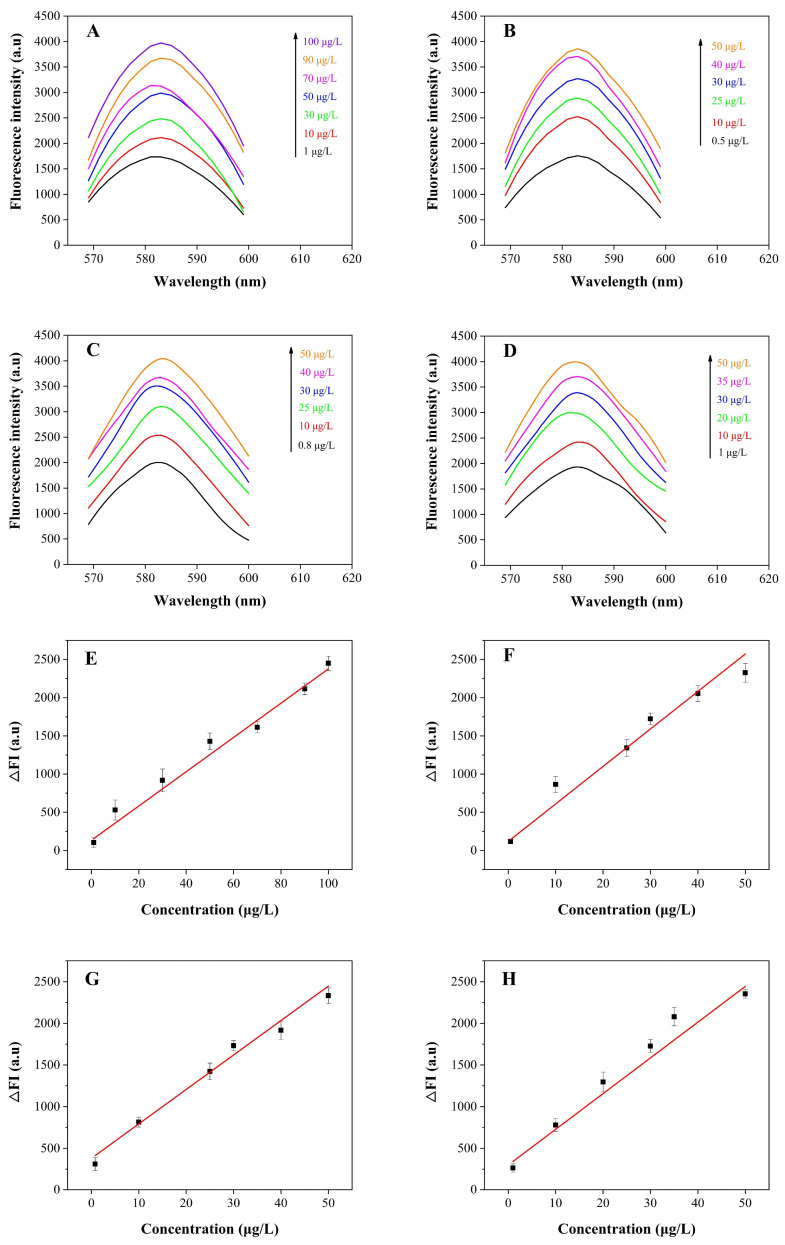
Fluorescence spectrums (excitation wavelength was 558 nm) of different concentrations of phorate (**A**), profenofos (**B**), isocarbophos (**C**), and omethoate (**D**). The linear relationships between recovered FI value (ΔFI) and targets concentrations (**E**), phorate; (**F**), profenofos; (**G**), isocarbophos; and (**H**), omethoate.

Under optimal conditions, we observed the fluorescence signal changes with OPs concentrations (Figure 6A–D). With increasing concentration of OPs target, the fluorescence spectrum intensity of TAMRA label gradually enhanced because the quenching effect by G bases was weakened. The standard calibration curves of the four kinds of OPs were constructed using TAMRA-Apt (20 nM) and cDNA15-G4 (60 nM). The linear relationships between the recovered fluorescence intensity (ΔFI) and different concentrations of phorate, profenofos, isocarbophos, and omethoate were shown, respectively, in Figure 6E–H. The regression coefficients (R^2^) were ranging from 0.9687 to 0.9847, the limits of detection (LODs) were in the range of 0.167 to 0.333 µg/L (S/N = 3). The linear equations, linear ranges, R^2^, and LODs were listed in Table 2.

**Figure 7:**
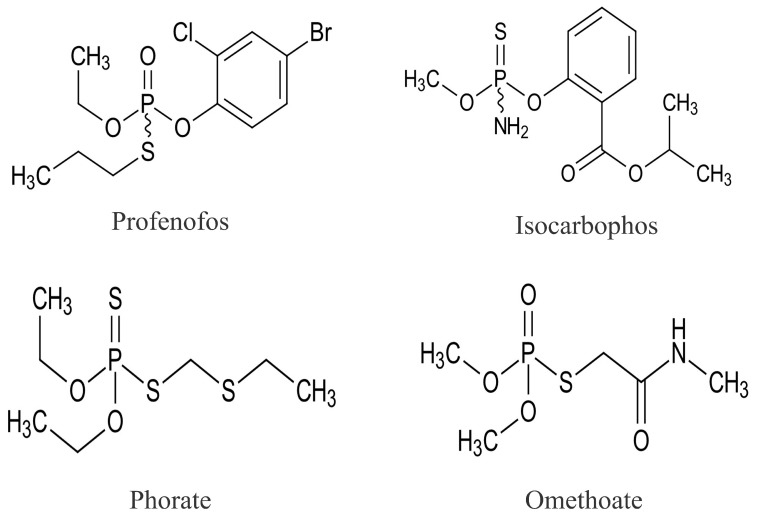
The molecular structures of the four OPs.

This detection method has different detection ranges and limits of detection for the four organophosphorus pesticides. The affinity between aptamer and target is mainly determined by the binding sites. The molecular structures of the four pesticides are shown in Figure 7. Phorate and isocarbophos contain P=S bonds, profenofos and omethoate contain P=O bonds, isocarbophos and omethoate contain C=O bonds, and so on. Aptamer specifically bind to organophosphorus pesticides by recognizing these specific chemical bonds. Furthermore, both profenofos and isocarbophos have a benzene ring structure, which may increase the binding sites of the aptamer. The limits of detection for these two pesticides are lower than that of phorate and omethoate.

Our fluorescence assay was carried out with only two DNA sequences, which was simpler and more rapid. Compared with other types of biosensors (aptamers or enzymes) and analysis methods that had been reported, this proposed strategy exhibited comparable or lower LODs for detecting pesticides (Table 3).

### 3.5. Selectivity of the Fluorescence Assay

**Figure 8:**
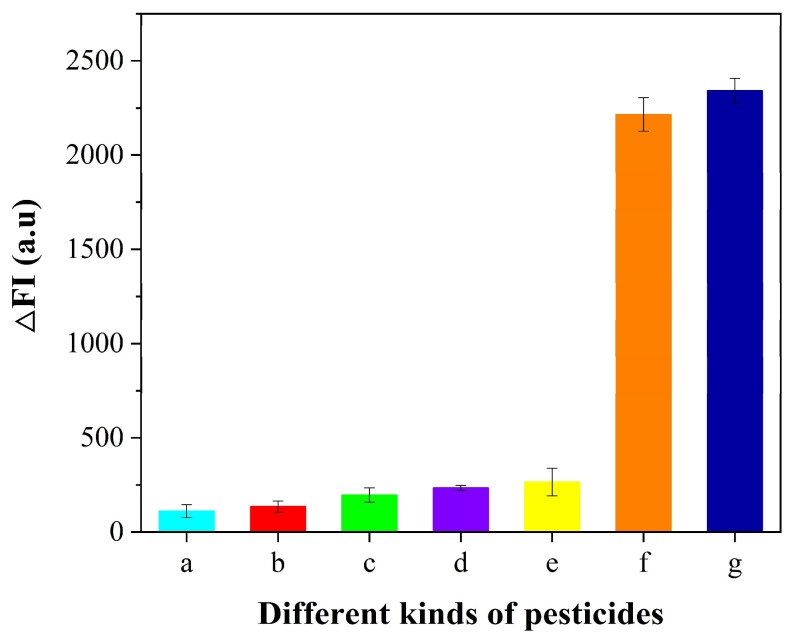
Selectivity tests of the fluorescence assay. (**a**) acetamiprid; (**b**) fenpropathrin; (**c**) malathion; (**d**) methyl parathion; (**e**) acetamiprid, fenpropathrin, malathion, methyl parathion; (**f**) phorate, profenofos, isocarbophos, omethoate; and (**g**) acetamiprid, fenpropathrin, malathion, methyl parathion, phorate, profenofos, isocarbophos, omethoate.

To verify the selectivity of our proposed assay for the specific OPs mentioned above, we tested four interfering pesticides (acetamiprid, fenpropathrin, malathion, and methyl parathion) in our fluorescence assay. We could see that when only interfering pesticides were present, there were no obvious ΔFI (Figure 8a–e). Figure 8f and g contained specific OPs targets (phorate, profenofos, isocarbophos, and omethoate) of the aptamer, and both of them had large ΔFI. Compared with Figure 8f (four specific OPs targets), there was no significant difference in Figure 8g (four specific OPs targets and four interfering pesticides). These results showed that the proposed method had good selectivity to the four kinds of OPs because the aptamer specifically bound with them.

### 3.6. Analysis of Actual Samples

In order to evaluate the performance of our fluorescence method, the standard addition method was used to determine the different concentrations of profenofos in vegetables. The vegetables were spiked at three levels (0, 30, and 90 µg/L) after LC confirmed that there were no pesticides residues. After extraction, the extracts diluted three times were determined by our method. The measured results were listed in Table 4, which showed that the recovery rate was ranging from 96.42% to 110.59%, and the RSD was between 4.09% and 11.23%. The above results suggested that the fluorescence method had great practicability for the detection of OPs in actual samples.

## 4. Conclusions

In summary, we developed a fluorescence method for the detection of four OPs (phorate, profenofos, isocarbophos, and omethoate), which used TAMRA-labeled aptamer and the complementary DNA with extended G bases. G near TAMRA quenched the fluorescence of TAMRA due to PET, and the presence of OPs target caused fluorescence recovery of TAMRA. Under optimized conditions, good linear relationships were established between fluorescence responses and analytes concentrations. This assay obtained low LODs, and combined merits of good selectivity, rapidity and simplicity. Moreover, satisfactory recovery of profenofos was achieved in the detection of actual samples. Therefore, this fluorescence strategy provided a reference for the detection of other pesticides or small molecules, using aptamers corresponding to the targets.

## Figures and Tables

**Table 1:** List of oligonucleotide sequences.

Name	Sequence (5′ to 3′)
TAMRA-Apt	TAMRA-AGCTTGCTGCAGCGATTCTTGATCGCCACAGAGCT
cDNA15	TCGCTGCAGCAAGCT
cDNA15-G4	TCGCTGCAGCAAGCTGGGG

**Table 2:** Main parameters for the fluorescence assay (*n* = 3).

Analyte	Linear Equation	Linear Range (µg/L)	R^2^	LOD (µg/L)
Phorate	ΔFI = 21.7 C + 218.24	1–100	0.9839	0.333
Profenofos	ΔFI = 43.7 C + 273.39	0.5–50	0.9719	0.167
Isocarbophos	ΔFI = 40.3 C + 375.57	0.8–50	0.9847	0.267
Omethoate	ΔFI = 44.1 C + 343.16	1–50	0.9687	0.333

**Table 3:** Comparison of the fluorescence assay with other types of methods for detecting pesticides.

Method	Pesticide	Linear Range (nM)	Detection Limit (nM)	Reference
Microcantilever-array	profenofos	13.38–2676.44	3.5	[35]
Fluorescence	isocarbophos	10–500	10	[36]
Gas chromatography	profenofos	267.64–13,382.22	89.21	[37]
	isocarbophos	345.67–17,283.69	115.22	
Electrochemistry	profenofos	0.01–100	0.003	[20]
	phorate	1–1000	0.3	
	isocarbophos	0.1–1000	0.03	
	omethoate	1–500	0.3	
Fluorescence	profenofos	268–26,800	134	[38]
	isocarbophos	346–34,600	35	
Fluorescence	profenofos	13.38–26,764.45	13.4	[33]
	isocarbophos	17.28–34,567.63	17.2	
	phorate	19.2–38,405.41	19.2	
	omethoate	23.45–46,906.12	23.4	
Fluorescence	phorate	3.84–384.05	1.28	Our assay
	profenofos	1.34–133.82	0.446	
	isocarbophos	2.77–172.84	0.922	
	omethoate	4.69–234.53	1.56	

**Table 4:** Recovery results of the proposed fluorescence assay in vegetables (*n* = 3).

Sample	Spiked (µg/L)	Diluted (µg/L)	Detected (µg/L)	RSD (%)	Recovery (%)
Lettuce	0	0	0	-	-
	30	10.516	31.548	4.91	105.16
	90	29.591	88.773	4.09	98.64
Cabbage	0	0	0	-	-
	30	11.059	33.177	11.23	110.59
	90	28.925	86.775	5.41	96.42

## Abbreviations

TAMRA	tetramethylrhodamine
cDNA	complementary DNA
G	guanine
PET	photoinduced electron transfer
OPs	organophosphorus pesticides
ACh	acetylcholine
MRL	maximum residue limit
MS	mass spectrometry
HPLC	high performance liquid chromatography
LC	liquid chromatography
GC	gas chromatography
FI	fluorescence intensity (emission wavelength at 583 nm)
LOD	limit of detection
R^2^	regression coefficient
RSD	relative standard deviation

## References

[1] Zhou J., Zou X., Song S., Chen G. (2018). Quantum dots applied to methodology on detection of pesticide and veterinary drug residues. J. Agric. Food Chem..

[2] Edwards C., Duanghathaipornsuk S., Goltz M., Kanel S., Kim D.S. (2019). Peptide nanotube encapsulated enzyme biosensor for vapor phase detection of malathion, an organophosphorus compound. Sensors.

[3] Jiang M., Chen C., He J., Zhang H., Xu Z. (2020). Fluorescence assay for three organophosphorus pesticides in agricultural products based on magnetic-assisted fluorescence labeling aptamer probe. Food Chem..

[4] Satnami M.L., Korram J., Nagwanshi R., Vaishanav S.K., Karbhal I., Dewangan H.K., Ghosh K.K. (2018). Gold nanoprobe for inhibition and reactivation of acetylcholinesterase: An application to detection of organophosphorus pesticides. Sens. Actuators B.

[5] Liu Y., Lv B., Liu A., Liang G., Yin L., Pu Y., Wei W., Gou S., Liu S. (2018). Multicolor sensor for organophosphorus pesticides determination based on the bi-enzyme catalytic etching of gold nanorods. Sens. Actuators B.

[6] Razmi H., Jabbari M. (2015). Development of graphene-carbon nanotube-coated magnetic nanocomposite as an efficient sorbent for HPLC determination of organophosphorus pesticides in environmental water samples. Int. J. Environ. Anal. Chem..

[7] Sgobbi L.F., Machado S.A.S. (2018). Functionalized polyacrylamide as an acetylcholinesterase-inspired biomimetic device for electrochemical sensing of organophosphorus pesticides. Biosens. Bioelectron..

[8] Dong J., Hou J., Jiang J., Ai S. (2015). Innovative approach for the electrochemical detection of non-electroactive organophosphorus pesticides using oxime as electroactive probe. Anal. Chim. Acta.

[9] Zhang S., Liu X., Qin J., Yang M., Zhao H., Wang Y., Guo W., Ma Z., Kong W. (2017). Rapid gas chromatography with flame photometric detection of multiple organophosphorus pesticides in Salvia miltiorrhizae after ultrasonication assisted one-step extraction. J. Chromatogr. B.

[10] Zhang A., Zhou L., Zhang P., Fan X., Wang C., Zhang L. (2012). Bamboo charcoal as solid-phase extraction adsorbent for the determination of organophosphorus pesticides in water samples by high-performance liquid chromatography. Anal. Lett..

[11] Zhao F., Wu J., Ying Y., She Y., Wang J., Ping J. (2018). Carbon nanomaterial-enabled pesticide biosensors: Design strategy, biosensing mechanism, and practical application. TrAC Trends Anal. Chem..

[12] Yao Y., Jiang C., Ping J. (2019). Flexible freestanding graphene paper-based potentiometric enzymatic aptasensor for ultrasensitive wireless detection of kanamycin. Biosens. Bioelectron..

[13] Guo X., Wen F., Qiao Q., Zheng N., Saive M., Fauconnier M.L., Wang J. (2019). A novel graphene oxide-based aptasensor for amplified fluorescent detection of aflatoxin M_1_ in milk powder. Sensors.

[14] Zhao R., Jia D., Wen Y., Yu X. (2017). Cantilever-based aptasensor for trace level detection of nerve agent simulant in aqueous matrices. Sens. Actuators B.

[15] Reinemann C., Fritsch U.F.V., Rudolph S., Strehlitz B. (2016). Generation and characterization of quinolone-specific DNA aptamers suitable for water monitoring. Biosens. Bioelectron..

[16] Gotrik M.R., Feagin T.A., Csordas A.T., Nakamoto M.A., Soh H.T. (2016). Advancements in aptamer discovery technologies. Acc. Chem. Res..

[17] Feng J., Dai Z., Tian X., Jiang X. (2018). Detection of Listeria monocytogenes based on combined aptamers magnetic capture and loop-mediated isothermal amplification. Food Control.

[18] Cheng S., Liu H., Zhang H., Chu G., Guo Y., Sun X. (2020). Ultrasensitive electrochemiluminescence aptasensor for kanamycin detection based on silver nanoparticle-catalyzed chemiluminescent reaction between luminol and hydrogen peroxide. Sens. Actuators B.

[19] Ni H., Zhang S., Ding X., Mi T., Wang Z., Liu M. (2014). Determination of enrofloxacin in bovine milk by a novel single-stranded DNA aptamer chemiluminescent enzyme immunoassay. Anal. Lett..

[20] Fu J., An X., Yao Y., Guo Y., Sun X. (2019). Electrochemical aptasensor based on one step co-electrodeposition of aptamer and GO-CuNPs nanocomposite for organophosphorus pesticide detection. Sens. Actuators B.

[21] Uniyal S., Sharma R. (2018). Technological advancement in electrochemical biosensor based detection of organophosphate pesticide chlorpyrifos in the environment: A review of status and prospects. Biosens. Bioelectron..

[22] Li X., Ping J., Ying Y. (2019). Recent developments in carbon nanomaterial-enabled electrochemical sensors for nitrite detection. Trac Trends Anal. Chem..

[23] Bala R., Kumar M., Bansal K., Sharma R.K., Wangoo N. (2016). Ultrasensitive aptamer biosensor for malathion detection based on cationic polymer and gold nanoparticles. Biosens. Bioelectron..

[24] Shi H., Zhao G., Liu M., Fan L., Cao T. (2013). Aptamer-based colorimetric sensing of acetamiprid in soil samples: Sensitivity, selectivity and mechanism. J. Hazard. Mater..

[25] Kang L., Yang B., Zhang X., Cui L., Meng H., Mei L., Wu C., Ren S., Tan W. (2015). Enzymatic cleavage and mass amplification strategy for small molecule detection using aptamer-based fluorescence polarization biosensor. Anal. Chim. Acta.

[26] Luo Q., Lai J., Qiu P., Wang X. (2018). An ultrasensitive fluorescent sensor for organophosphorus pesticides detection based on RB-Ag/Au bimetallic nanoparticles. Sens. Actuators B.

[27] Torimura M., Kurata S., Yamada K., Yokomaku T., Kamagata Y., Kanagawa T., Kurane R. (2001). Fluorescence-quenching phenomenon by photoinduced electron transfer between a fluorescent dye and a nucleotide base. Anal. Sci..

[28] Heinlein T., Knemeyer J.P., Piestert O., Sauer M. (2003). Photoinduced electron transfer between fluorescent dyes and guanosine residues in DNA-hairpins. J. Phys. Chem. B.

[29] Zhao Q., Tao J., Feng W., Uppal J.S., Peng H., Le X.C. (2020). Aptamer binding assays and molecular interaction studies using fluorescence anisotropy-a review. Anal. Chim. Acta.

[30] Wang X., Yang Y., Yin Y., Zeng N., Dong Y., Liu J., Wang L., Yang Z., Yang C. (2022). High-throughput aptamer microarrays for fluorescent detection of multiple organophosphorus pesticides in food. Anal. Chem..

[31] Li Y., Zhao Q. (2020). Aptamer structure switch fluorescence anisotropy assay for aflatoxin B1 using tetramethylrhodamine-guanine interaction to enhance signal change. Chin. Chem. Lett..

[32] Liu L., Zhao Q. (2020). A simple fluorescence anisotropy assay for detection of bisphenol A using fluorescently labeled aptamer. J. Environ. Sci..

[33] Zhang C., Wang L., Tu Z., Sun X., He Q., Lei Z., Xu C., Liu Y., Zhang X., Yang J. (2014). Organophosphorus pesticides detection using broad-specific single-stranded DNA based fluorescence polarization aptamer assay. Biosens. Bioelectron..

[34] Zhang D., Shen H., Li G., Zhao B., Yu A., Zhao Q., Wang H. (2012). Specific and sensitive fluorescence anisotropy sensing of guanine-quadruplex structures via a photoinduced electron transfer mechanism. Anal. Chem..

[35] Li C., Zhang G., Wu S., Zhang Q. (2018). Aptamer-based microcantilever-array biosensor for profenofos detection. Anal. Chim. Acta.

[36] Li X., Tang X., Chen X., Qu B., Lu L. (2018). Label-free and enzyme-free fluorescent isocarbophos aptasensor based on MWCNTs and G-quadruplex. Talanta.

[37] Wang P., Luo M., Liu D., Zhan J., Liu X., Wang F., Zhou Z. (2018). Application of a magnetic graphene nanocomposite for organophosphorus pesticide extraction in environmental water samples. J. Chromatogr. A.

[38] Dou X., Chu X., Kong W., Luo J., Yang M. (2015). A gold-based nanobeacon probe for fluorescence sensing of organophosphorus pesticides. Anal. Chim. Acta.

